# Collodion as a Coating for Pills

**Published:** 1849-05

**Authors:** E. H. Durden


*Collodion as a coating for Pills.* By Mr. E. H. Durden.—I find that collodion may be usefully employed for coating pills; for this purpose the pills may be placed on the point of a fine pin or needle, and dipped into the solution of gun cotton. The solution I used had a sp. gr. of 0.810, and I found two dippings gave a perfect coating. An aloetic or colocynth pill thus covered may be placed on the tongue, and no taste experienced of the bitter ingredients entering into its composition. Its medicinal effect is not interfered with. Solutions of gutta percha in chloroform, and bisulphuret of carbon, effects the same purpose, but the collodion is preferable.—*Pharmaceutical Journal.*

